# Transcriptome-Wide Analysis of Human Liver Reveals Age-Related Differences in the Expression of Select Functional Gene Clusters and Evidence for a PPP1R10-Governed ‘Aging Cascade’

**DOI:** 10.3390/pharmaceutics13122009

**Published:** 2021-11-25

**Authors:** Thomas Schreiter, Robert K. Gieseler, Ramiro Vílchez-Vargas, Ruy Jauregui, Jan-Peter Sowa, Susanne Klein-Scory, Ruth Broering, Roland S. Croner, Jürgen W. Treckmann, Alexander Link, Ali Canbay

**Affiliations:** 1 Department of Medicine, University Hospital Knappschaftskrankenhaus Bochum, Ruhr University Bochum, 44892 Bochum, Germany; thomas.schreiter@ruhr-uni-bochum.de (T.S.); rk.gieseler@gmx.de (R.K.G.); jan.sowa@ruhr-uni-bochum.de (J.-P.S.); susanne.klein-scory@ruhr-uni-bochum.de (S.K.-S.); 2 Laboratory of Immunology & Molecular Biology, University Hospital Knappschaftskrankenhaus Bochum, Ruhr University Bochum, 44892 Bochum, Germany; 3 Department of Gastroenterology, Hepatology, and Infectious Diseases, Medical Faculty, Otto-von-Guericke University, 39120 Magdeburg, Germany; ramiro.vilchez@med.ovgu.de (R.V.-V.); Alexander.Link@med.ovgu.de (A.L.); 4 Data Science Grasslands, Grasslands Research Centre, AgResearch, Palmerston North 4410, New Zealand; Ruy.Jauregui@agresearch.co.nz; 5 Department of Gastroenterology and Hepatology, University Hospital Essen, University of Duisburg-Essen, 45147 Essen, Germany; Ruth.Broering@uk-essen.de; 6 Department of General, Visceral, Vascular and Transplantation Surgery, Medical Faculty, Otto-von-Guericke University, 39120 Magdeburg, Germany; roland.croner@med.ovgu.de; 7 Department of General, Visceral and Transplantation Surgery, University Hospital Essen, University of Duisburg-Essen, 45147 Essen, Germany; juergen-walter.treckmann@uk-essen.de; 8 Section of Hepatology and Gastroenterology, University Hospital Knappschaftskrankenhaus Bochum, Ruhr University Bochum, 44892 Bochum, Germany

**Keywords:** aging, EFS, hepatocellular carcinoma, inflammaging, liver regeneration, liver transcriptome, non-alcoholic fatty liver disease, non-alcoholic steatohepatitis, pharmacogenes, regulome

## Abstract

A transcriptome-wide analysis of human liver for demonstrating differences between young and old humans has not yet been performed. However, identifying major age-related alterations in hepatic gene expression may pinpoint ontogenetic shifts with important hepatic and systemic consequences, provide novel pharmacogenetic information, offer clues to efficiently counteract symptoms of old age, and improve the overarching understanding of individual decline. Next-generation sequencing (NGS) data analyzed by the Mann–Whitney nonparametric test and Ensemble Feature Selection (EFS) bioinformatics identified 44 transcripts among 60,617 total and 19,986 protein-encoding transcripts that significantly (*p* = 0.0003 to 0.0464) and strikingly (EFS score > 0.3:16 transcripts; EFS score > 0.2:28 transcripts) differ between young and old livers. Most of these age-related transcripts were assigned to the categories ‘regulome’, ‘inflammaging’, ‘regeneration’, and ‘pharmacogenes’. NGS results were confirmed by quantitative real-time polymerase chain reaction. Our results have important implications for the areas of ontogeny/aging and the age-dependent increase in major liver diseases. Finally, we present a broadly substantiated and testable hypothesis on a genetically governed ‘aging cascade’, wherein *PPP1R10* acts as a putative ontogenetic master regulator, prominently flanked by *IGFALS* and *DUSP1*. This transcriptome-wide analysis of human liver offers potential clues towards developing safer and improved therapeutic interventions against major liver diseases and increased insights into key mechanisms underlying aging.

## 1. Introduction

According to present-day criteria, the script for the evolutionarily founded dramaturgy of the individual lifetime of early *Homo sapiens* lacks a chapter for old age: The average life expectancy of Paleolithic hunter–gatherers from the appearance of *Homo sapiens* ~200,000 years ago was ~30 years [1]. While there was an increase in longevity in modern humans of the Early Upper Paleolithic ~50,000 years ago [2], our species has only today arrived at an average global life expectancy of 72.81 years (United Nations figure for 2021). Centenarians are rare, and the longest confirmed lifespan among supercentenarians is 122 years [3]. In the distant past, an individual thus left the stage long before entering old age as we understand it today. The genetic underpinnings of humans were therefore shaped on the requirement to optimize and adapt biological functions for a phase of life nowadays considered as young. Current lifespans were out of reach and their needs, therefore, biologically irrelevant. This bequeathed today’s humans with genetic hardware whose regulation and copyediting in progressed age increasingly suffer from error and inaccuracy, thus entailing age-related disease and premature demise. Our work joins previous efforts to deal with this imperfect legacy.

With its well-known altruistic function, but also fending for itself, the liver stands out as an organ of major systemic importance. This justifies the approach of a whole-transcriptome analysis of the human liver, with the objective to reveal decisive differences between young and old transcriptomes. Aiming to identify all functional elements in the human genome [4], the ENCODE Project could in 2012 assign functions to ~80% of the genome in particular outside of well-studied protein-coding regions, including sequence variants linked to human disease [5]. Similarly, it was interesting for us to see whether previously inapparent transcripts stand out prominently upon age-related liver transcriptome analysis. We thus for the first time identified the most salient aging-related differences in the liver transcriptome. Data relevance was validated by statistical significance and a bioinformatics approach determining Ensemble Feature Selection (EFS) scores [6].

Importantly, mRNA translation rates prognosticate associated protein levels with median correlations of ~0.9 between predictions and measurements [7]. Doubts about the soundness of this reasoning [8] were refuted convincingly [9]. Thus, our findings on the liver transcriptome can serve for harnessing their inherent opportunities to better address hepatological, pharmacological, and geriatric needs, as well as to gain further insight into the overarching topic of aging. Data were assigned to distinct functional categories, where most important age-dependent differences were found in the ‘regulome’ [10,11] category and the ‘regulome–regeneration–inflammaging’ intersection. Certain genetic components of the regulome thus are of major biological relevance with regard to individual aging. They were harmonized with an ‘axis of aging’ characterized previously [12,13] to be expanded into a regulatory ‘aging cascade’ in our present article.

## 2. Patients, Materials and Methods

### 2.1. Liver Tissue

The study protocol conformed to the revised 2008 Declaration of Helsinki and was approved by the Ethics Committees of the University Hospitals Essen (file number: 12-5232-BO; 6 December 2012) and Magdeburg (file number: 208/17; 8 January 2018). Liver tissue samples were collected during the periods of 2013–2017 (Essen) or 2018–2019 (Magdeburg), respectively.

All patients were of Caucasian descent. They provided informed consent before undergoing partial liver resections due to the underlying diseases specified in Table S1. This table also provides further demographic information and clinical characteristics, including comorbidities and further information gathered from the patients’ files.

According to standard good practice, the major portions of resected tissue—including both diseased as well as surrounding healthy tissue—were examined by the pathologist. For the present study, we therefore only investigated liver tissue samples from the periphery of resected tissue that, because of unimpaired appearance and structure, were considered as ‘normal’. After quality inspection, liver tissue samples eventually underwent the methodologic steps required for enabling transcriptome-wide analysis (see below).

### 2.2. RNA Preparation

Tissue was homogenized in 1 mL of Trizol using the QIAshredder equipment (Qiagen, Hilden, Germany), and RNA was isolated as described before [14]. Pellets were resuspended in 100 µL of nuclease-free water and purified using the RNeasy Mini Kit (Qiagen). RNA was eluted with 50 µL of nuclease-free water. Concentration and purity were determined with the NanoDrop One spectrophotometer (Thermo Fisher, Dreieich, Germany). Samples were treated with DNAse (Turbo DNA-free kit (Ambion/Invitrogen, Thermo Fisher)) at 37 °C for 60 min. RNA was precipitated overnight with ethanol containing sodium acetate at −80 °C. Precipitates were collected by centrifugation (30 min, 17,000× *g*, 4 °C). Pellets were dried for 30 min at RT and resuspended in 50 µL nuclease-free water (or 30 µL in case that the amount of RNA measured before DNase digestion was <200 ng/µL) according to the amount of RNA measured before DNase digestion. For confirmation or correction, RNA concentrations and purities were determined again (cf. above). Samples were stored at –80 °C until processing.

### 2.3. RNA Quality Assessment

Using 20 µL preparations, each, of DNA-free RNA from 37 patients, the RNA quality number (RQN) for each RNA preparation was determined using the Qubit system (Thermo Fisher) and by electrophoretic separation. While an RQN > 7 was considered suitable for sequencing, slightly lower RQNs (minimum: 6.2) were considered sufficient in the absence of degradation evidenced by chromatography. Subsequently, next-generation sequencing was performed.

### 2.4. Next-Generation Sequencing (NGS)

RNA obtained from homogenized liver tissue samples was subjected to NGS. Samples were assigned to Group I (Young; <49 years. (23–48, median: 34.3); 3 males, 6 females) and Group II (Old: >74 years. (75–79, median: 78.6); 6 males, 2 females) (cf. Table S1). Transcriptome-wide analysis revealed a total of 60,617 RNA transcripts. Counts ranged from 1.156–1.865 × 10^7^ (Group I) and 1.020–2.080 × 10^7^ (Group II). In transcriptome profiling by RNA sequencing, the number of mapped reads for a given gene depends on its expression level, length, and sequencing depth. Thus, transcript expression levels are normalized as transcripts per million (TPM), which represent the relative transcript abundance among a population of sequenced transcripts [15]. We defined values of >50 TPM for ≥1/group as relevant for further investigation (see also the Results section).

### 2.5. Quantitative Real-Time Polymerase Chain Reaction (qRT-PCR)

Remainders of the RNA preparations applied to NGS were adjusted to 125 ng/µL. RNA at 1 µg per sample was transcribed to cDNA using the QuantiNova Reverse Transcription Kit (Qiagen, Hilden, Germany) in a Mastercycler gradient (Eppendorf, Hamburg, Germany) at 25 °C (3 min), 45 °C (12 min), 85 °C (5 min), and 20 °C (2 min). The reaction volume of 20 µL was diluted to 10 ng/µL by adding 80 µL of nuclease-free water and stored at −20 °C. Wet lab-validated primers from the QuantiNova LNR PCR assay (Qiagen) were deployed for the following targets given in alphabetical order: *AGO2*, *CFLAR*, *CYP3A43*, *DUSP1*, *EGR1*, *FAH*, *FLNA*, *GATA4*, *HSD17B14*, *IGFALS*, *ITSN1*, *KIAA0040*, *LIPC*, *PALLD*, *PPP1R10*, and *TFF3*, as well as *PPIA* as a reference (for sequences, see Table S2). QRT-PCRs were performed in a LightCycler 480 (Roche Diagnostics, Rotkreuz, Switzerland) with the QuantiNova SYBR Green PCR Kit (Qiagen) in reaction volumes of 18 µL/well containing 2 µL (20 ng) template, 2 µL probe assay (10× primer or reference), 9 µL QuantiNova probe master mix, and 5 µL RNase-free water. Amplification was executed at 95 °C (2 min), with 45 cycles at 95 °C (5 s, each), 60 °C (10 s, each), and 70 °C (30 s), with the melting curve from 70 °C to 95 °C in 50 cycles increasing by 0.5 °C/s per cycle. Differences in relative gene expressions were calculated from the crossing point (Cp) values for Groups I and II:

$$Fold\;expression=\frac{2^{Target\;Group\;II\;-\;Reference\;Group\;II}}{2^{Target\;Group\;I\;-\;Reference\;Group\;I}}$$

### 2.6. Data Analyses

#### 2.6.1. Statistics

Statistical analyses were performed with Microsoft Office Excel 2019 (Microsoft, Redmond, WA, USA) and Graph Pad 8.0 (GraphPad Software, La Jolla, CA, USA). Differences of gene expressions between Groups I and II were calculated in MS Excel by the Mann-Whitney non-parametric test using the Real Statistics Resource Pack add-on, which is available at http://www.real-statistics.com (accessed on 4 March 2021). Significant *p*-values (<0.05) of 257 genes were confirmed with Graph Pad 8.0.

#### 2.6.2. EFS

Feature selection methods are part of the development of classification strategies within the supervised learning area of artificial intelligence. They are often used to address the problem of identifying characteristic subsets of features present in large and complex datasets by ranking features in order of importance, and by allowing for the elimination of features irrelevant to the classification question, thus reducing the dimensionality and complexity of the data [16]. EFS addresses limitations identified by the use of single feature selection strategies often used in medical modeling (such as the Gini index), which have been shown to produce unstable results [17] by the aggregation of multiple feature selection methods. This strategy has been successfully applied to direct oncogene discovery from cancer screening data [18], predict metastasis [19], and identify early sepsis molecular signatures [20]. We here applied the EFS method as described in [6], where eight different Single Feature selection methods are combined, with their outputs normalized to avoid biases inherent to any single method. EFS software can be downloaded free of charge as an R-package from the Comprehensive R Archive Network (CRAN) [6] or, alternatively, used as a web application at http://EFS.heiderlab.de (accessed on 7 March 2021). ‘R’ stands for the programming language, i.e., R ≥ 3.0.2.

### 2.7. Data Availability

All raw and processed sequencing data generated in this study have been submitted to the NCBI Gene Expression Omnibus (GEO; https://www.ncbi.nlm.nih.gov/geo/, accessed on 7 March 2021). The datasets and the computer code produced in this study are available in the following databases: https://www.ncbi.nlm.nih.gov/geo/query/acc.cgi?acc=GSE183915 accessed on 7 March 2021.

## 3. Results and Discussion

### 3.1. Methodology and Strategy

**Figure 1:**
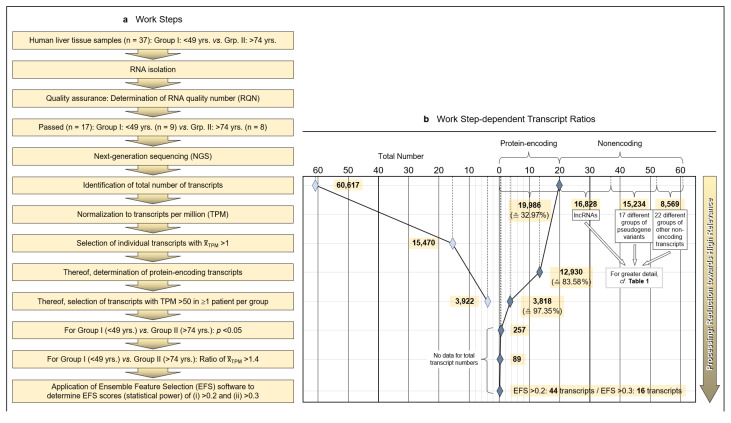

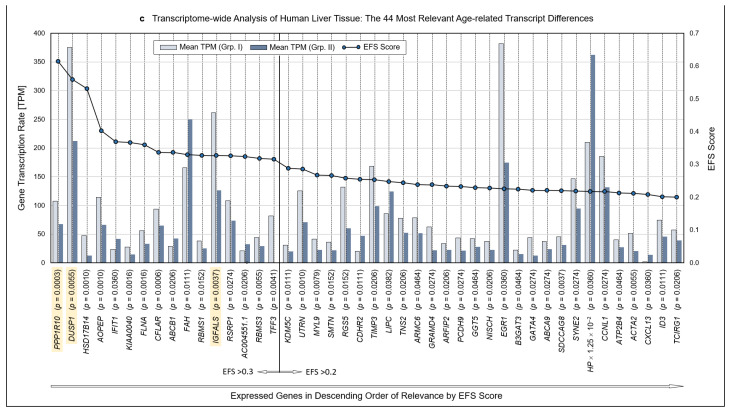
Methodology and strategy for transcriptome-wide analysis of human liver tissue, resulting protein-encoding and non-encoding transcripts, and most relevant age-related transcript differences. (**a**) Flow diagram of the works steps from the initial sampling of human liver tissue and the detection of a total of 60,617 hepatic RNA transcripts down to the eventual EFS software-supported pinpointing of 16 gene transcripts of major interest related to their different expressions in age Groups I and II. (**b**) Diagram depicting the ratios upon narrowing down the complete transcriptome of the human liver to the most relevant age-related differences in hepatic gene expression, with Ensemble Feature Selection (EFS) scores of >0.2 or >0.3, respectively. Step-wise results are even-leveled with the respective work steps in panel (**a**) and are differentiated between total transcripts (left) and protein-encoding vs. non-encoding RNAs (right). After selecting for >50 transcripts per million (TPM) (cf. panel (**a**)), only protein-encoding transcripts were determined. Transcripts initially enumerated comprised 19,986 protein-encoding and 40,631 nonencoding RNAs, with the latter differentiated as shown in panel (**b**) and further broken down into their biotypes in Table 1. (**c**) Comparisons of the 44 mean gene transcription rates between Groups I and II resulting from the final processing step in panel (**b**), aligned in sequence of descending EFS scores.

Tumor-surrounding healthy areas of resected tissue from patients undergoing surgical intervention for malignancy were processed for whole-transcriptome analysis to investigate age-dependent differences between protein-encoding mRNAs. As detailed in the Patients and Methods section, isolated and quality-assured RNA was subjected to next-generation sequencing (NGS). Total transcripts were normalized to transcripts per million (TPM), and protein-encoding transcripts were identified and narrowed down to transcripts of high age-dependent (Group I: <40 years; Group II: >74 years) relevance by applying both the Mann–Whitney nonparametric test and EFS software generating EFS scores (Figure 1a).

### 3.2. Limitation of Gene Expression to Exceptional Age-Dependent Differences

Initially, a total number of 60,617 hepatic transcripts was identified, which included 19,986 protein-encoding transcripts. Employing the step-wise procedure sketched in Figure 1a, this number was narrowed down to 44 transcripts of high (EFS scores > 0.2) and 16 transcripts of outstanding relevance (EFS scores > 0.3) as to their age-dependent differences between Groups I (Young) and II (Old) (Figure 1b; Table S3). Note that after selecting for >50 TPM, only protein-encoding transcripts were determined. Table 1 details the biotypes of the initially detected transcripts.

### 3.3. Identification of Transcripts Displaying High Age-Dependent Differences

Identified RNAs are aligned in sequence of descending EFS scores, further mentioning the *p* values of the transcripts’ age-dependent differences (Figure 1c; the genes highlighted therein will be referred to below). For the complete set of associated data, see Table S3. Further accompanying information is presented in Table S4. As detailed therein, ≥3 of the high-relevance transcripts, each, accumulated on chromosomes 1, 3, 5, 11, and 22; these associations may spur further research as to whether certain genes or groups of genes might be transcriptionally co-regulated.

### 3.4. Functional Categorization of Selected Transcripts

**Figure 2:**
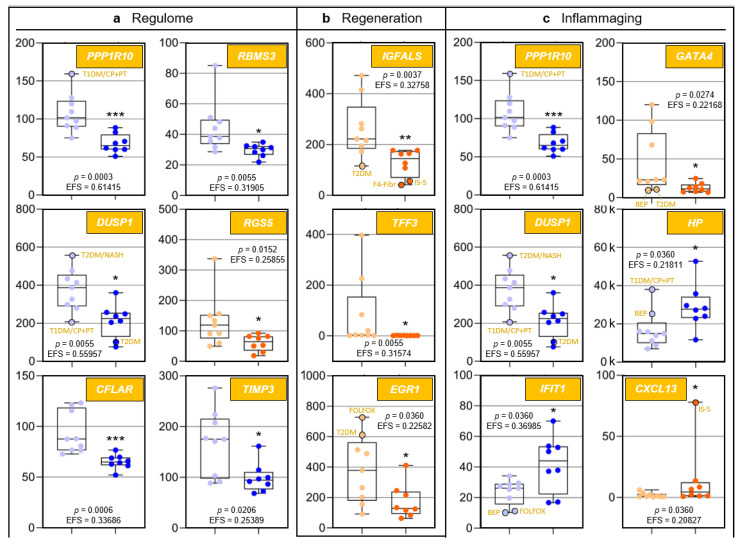

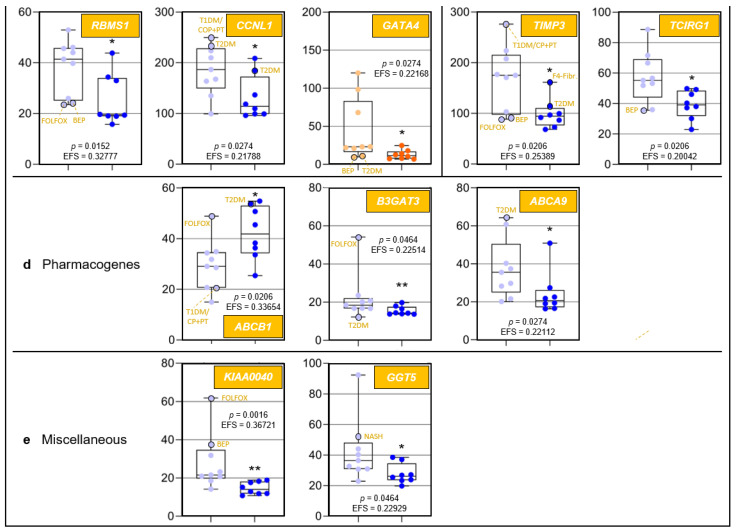
Age-related differences of transcripts assigned to regulome, inflammaging, liver regeneration, and pharmacogenes, as well as non-assigned hepatic transcripts. In each category, data are depicted in sequence of their relevance determined by Ensemble Feature Selection (EFS). Each graph compares Group I/Young (left) to Group II/Old (right). Where ratios between Groups I and II differ by at least a factor of × 2, data are depicted in red; all other data are shown in blue (cf. Table S3). Results are expressed as means ± standard deviation (SD) in the form of box and whisker plots. Significances between Group I vs. II transcript expressions range from *p* = 0.0464 to *p* = 0.0006 (i.e., *, **, or ***, respectively). If relevant, graphs are represented in different categories. (**a**) Regulome: CASP8- and FADD-like apoptosis regulator (*CFLAR*); RNA-binding motif single-stranded interacting protein 1 (*RBMS1*); RNA-binding motif single-stranded interacting protein 3 (*RBMS3*); and regulator of G protein signaling 5 (*RGS5*). (**b**) Regeneration: insulin-like growth factor-binding protein acid-labile subunit (*IGFALS*), trefoil factor 3 (*TFF3*), early growth response 1 (*EGR1*), and cyclin L1 (*CCNL1*). (**c**) Inflammaging: interferon-induced protein with tetratricopeptide repeats 1 (*IFIT1*); tissue inhibitor of metalloproteinase 3 (*TIMP3*); GATA-binding protein 4 (*GATA4*); C-X-C motif chemokine ligand 13 (*CXCL13*); and T-cell immune regulator 1, ATPase H^+^ transporting V0 subunit a_3_ (*TCIRG1*). (**d**) Pharmacogenes (we not only include enzymes, but also systemic and cellular drug transporters into the definition of this term): ATP-binding cassette subfamily B member 1 (*ABCB1*); β-*1,3*-glucuronyltransferase 3 (*B3GAT3*); ATP-binding cassette subfamily A member 9 (*ABCA9*); and haptoglobin (*HP*). (**e**) Miscellaneous: The unknown protein KIAA0040 (*KIAA0040*); and γ-glutamyltransferase 5 (*GGT5*). For an integrated systematic representation and classification of the findings, see Figure 3. For additional information, we encircled datapoints (black) denoting extremes or outliers potentially associated with certain treatment regimens or comorbidities, respectively. Abbreviations (orange lettering) for preoperative treatments in context with the indications requiring partial liver resection or for diseases in addition to those indications: CP/PT, carboplatin + paclitaxel; F4-Fibr, fibrosis grade 4; FOLFOX, folinic acid + fluorouracil + oxaliplatin; IS-S, in-situ split (preoperatively); BEP, bleomycin + etoposide + cisplatin; NASH, non-alcoholic steatohepatitis; T1DM, type 1 diabetes mellitus; T2DM, type 2 diabetes mellitus.

**Figure 3:**
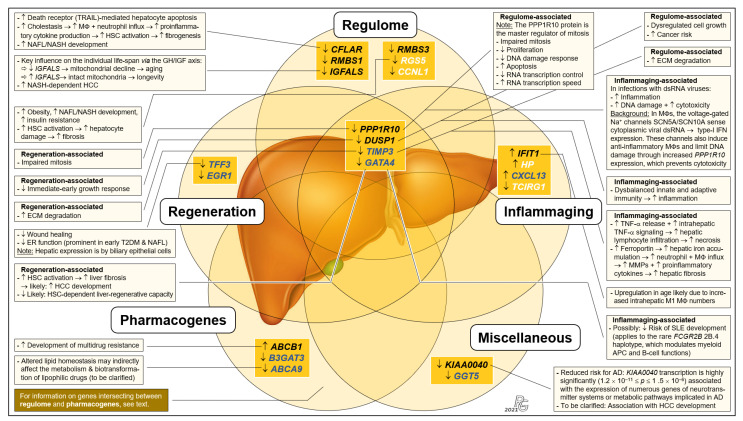
Integrated systematic representation and classification of the age-dependent expression of the liver transcriptome. Transcripts assigned to the categories ‘regulome’, ‘inflammaging’, ‘liver regeneration’, ‘pharmacogenes’, and ‘miscellaneous’ are depicted as overlapping sets. In each set, transcribed genes are listed in sequence of their EFS scores. The singular ‘regulome’ category and the ‘regulome–liver regeneration–inflammaging’ intersection include the genes *IGFALS* as well as *PPP1R10* and *DUSP1*, respectively, which are proposed as key elements of an ‘aging cascade’ discussed below and depicted in Figure 4. According to age-related differences, transcripts are either most relevant (EFS > 0.3; black font), display broader variances in Group I (cf. Figure 2) (EFS > 0.2; blue), or are other relevant transcripts (EFS > 0.2; white). Transcript changes in Group II vs. Group I are indicated as downregulated (↓) or upregulated (↑), and the most important consequences in aged individuals are characterized as downregulated/decreased (↓), upregulated/increased (↑), or process/consequence (→) (see text for greater detail). For gene abbreviations, cf. legend to Figure 2. Further abbreviations: AD, alcohol dependence; APC, antigen-presenting cell; dsRNA, double-stranded RNA; ECM, extracellular matrix; ER, endoplasmic reticulum; HSC, hepatic stellate cell; IFN, interferon; M1, M2, pro- and anti-inflammatory MΦ polarization states; MMP, matrix metalloproteinases; MΦ, macrophage; T2DM, type-2 diabetes mellitus; TNF-α, tumor necrosis factor α; TRAIL, TNF-related apoptosis-inducing ligand (a death receptor).

Because to their functional profiles, 25 of the 44 protein-encoding transcripts displayed in Figure 1c were assigned to the categories ‘regulome’, ‘regeneration’, ‘inflammaging’, ‘pharmacogenes’, and ‘miscellaneous’ (Figure 2 and Figure 3) and to the prominent intersections ‘regulome-regeneration’ and ‘regulome-inflammaging’ (Figure 3). These categories are the basis for expanding below on the general age-related implications, disease-specific associations, and overarching ontogenetic importance of these hepatic transcripts.

### 3.5. Confirmation of NGS Results by qRT-PCR

In order to confirm/verify the whole-transcriptome data of human liver, we performed qRT-PCR analyses employing a selection of transcripts from the high-relevance RNAs, plus a set of control transcripts. As a result, the transcript ratios obtained by NGS for the high-relevance RNAs were generally confirmed by the ‘fold-change’ data determined via qRT-PCR. Only the control transcripts of the genes encoding for fumarylacetoacetate hydrolase (*FAH*) and for lipase C (*LIPC*) revealed contrary results (Table 2).

### 3.6. Interpretations and Implications Related to Functional Gene/Transcript Categories

To our knowledge, this is the first whole-transcriptome analysis of human liver aiming at identifying major age-dependent differences in gene expression. However, due to the liver’s overarching role and importance, hepatic gene expressions must also be viewed in the systemic context. Here, the regulome comes into play, which comprises all regulatory components in health and disease [10,11,21,22]. Discussing the major implications of our findings concerning liver regeneration, inflammaging, pharmacogenes, and certain diseases clearly highlights the overall importance of distinct ‘regulome’-assigned transcripts.

#### 3.6.1. ‘Regeneration’-Assigned Transcripts

In humans, the liver is the only solid organ using self-regenerative mechanisms enabled by hepatocytes and cholangiocytes that can transdifferentiate into each other as facultative stem cells [23]. We assigned the transcripts of the genes protein phosphatase 1 regulatory subunit 10 (*PPP1R10*) (also known as phosphatase 1 nuclear targeting subunit (*PNUTS*)), dual specificity phosphatase 1 (*DUSP1*) (also known as map kinase phosphatase-1), and TIMP metallopeptidase inhibitor 3 (*TIMP3*) to the ‘regulome–regeneration’ intersection (Figure 3). While no studies have been performed on the contribution of *PPP1R10* to liver regeneration, its central function in this category is obvious from its key role as a master regulator of mitosis [24,25,26,27]. *DUSP1*, an immediate-early growth response gene, is activated in liver regeneration [28] and upregulated 1–2 h after liver injury [29]. As for *TIMP3*, liver regeneration requires the restoration of cellular mass and extracellular matrix (ECM) remodeling: Matrix metalloproteinases (MMPs) and their TIMP inhibitors regulate ECM turnover as well as growth factor and cytokine processing. *TIMP3* is significantly induced during liver regeneration [30]; its complete loss in age thus entails increased ECM degradation. Hepatic expression of trefoil factor 3 (*TFF3*) by biliary epithelial cells contributes to their migration and proliferation in wound healing [31,32]. Approximately 50% of the individuals in Group I showed different degrees of upregulated *TFF3* expression demonstrating ongoing regeneration, while complete downregulation in Group II indicates that hepatic regeneration requirements are underserved in age. Moreover, unfolded protein accumulation in the endoplasmic reticulum (ER) causes ER stress. Complete *TFF3* silencing linked to impaired ER function is prominent in early type-2 diabetes mellitus (T2DM) and hepatic steatosis [33]. *TFF3* modulation might thus be a future option for antagonizing ER stress and support liver regeneration in older people with non-alcoholic fatty liver disease (NAFLD). Collectively, significant downregulation of certain ‘regeneration’-assigned transcripts indicates a considerably impaired liver-regenerative capacity in age.

#### 3.6.2. ‘Inflammaging’-Assigned Transcripts

Inflammaging [34] entails greater disease susceptibility, increased morbidity/mortality [34,35], and frailty [36]. Significantly increased IL-6, TNF-α, and the acute-phase pentraxin, CRP (exclusively produced by hepatocytes [37]) are associated with frailty [35]. The transcripts assigned to the categories ‘regulome–inflammaging’ and ‘inflammaging’ (Figure 3) are a rich source for novel insight into inflammaging: In human macrophages (MΦs), the *SCN5A* and *SCN10A* splice variant products of a voltage-gated Na^+^ channel gene sense viral dsRNA, which upregulates the expression of type-I interferons. The channel prevents cytotoxicity via increased *PPP1R10* expression, which polarizes MΦs to an anti-inflammatory phenotype [38]. In age, upregulated inflammation in certain viral infections due to strongly reduced *PPP1R10* expression thus may contribute to inflammaging. The DUSP1 protein is a central regulator of both innate and adaptive immunity, and altered *DUSP1* expression is implicated in chronic inflammation [39,40,41]. Profoundly downregulated *DUSP1* expression in age thus suggests a prominent role in inflammaging. *TIMP3* expression is intimately connected with the release of TNF-α and IL-6: Specifically, rapid and powerful anti-infectious immunity is, inter alia, enabled by the shedding of preformed membrane-bound TNF-α [42], which is inhibited by TIMP3. Loss of *TIMP3* leads to hepatic lymphocyte infiltration and necrosis [43]. In *TIMP3*^−/−^ mice, enhanced TNF-α signaling is indicated by elevated serum IL-6 [42]. Therefore, age-dependently reduced *TIMP3* expression may underly the significant increase in TNF-α and IL-6 in inflammaging and frailty [35]. Finally, evidence supports a key role of increased *IFIT1* expression in inflammaging: Proinflammatory proteins induced in M1 MΦs prominently include gene products transcriptionally controlled by interferon regulatory factor 1, which strongly upregulates *IFIT1/-2/-3* expression [44]. Indeed, both M1 and M2 MΦ numbers increase in the aged liver [45]. IFIT1 also strongly interferes with viral mRNA translation, propagation, and pathogenicity [46,47,48]. While increased *IFIT1* expression in age thus might suggest improved antiviral immunity, overall anti-infective immunity actually erodes in aging [49,50], which entails more frequent and severe viral infections [51]. However, as age-related deteriorated antiviral immunity is due to defective T cell responsivity [52], increased *IFIT1* expression might be a compensatory mechanism. The fact that inflammaging in (super)centenarians is always counteracted by anti-inflammaging mechanisms may be one of the secrets underlying longevity [53].

#### 3.6.3. ‘Pharmacogene’-Assigned Transcripts

The Pharmacogenomics Knowledgebase (PharmGKB): https://www.pharmgkb.org (accessed on 20 August 2021) comprises a set of ‘very important pharmacogenes’ (VIPs) [54]. However, its underlying data have been determined in young adults, while age-dependent changes profoundly influence VIP expressions and, therefore, the efficacy and biotransformation of pharmaceuticals. Striking differences vs. adults were demonstrated in fetuses [55], children, and adolescents [56], so that alterations are expected to occur throughout individual development, including in old age. For example, drug-induced liver injury in age is most likely caused by antibiotics and cardiovascular drugs [57]. Moreover, pharmacogenes are not limited to the PharmGKB’s current VIP set: We show that the differences in the transcription of *PPP1R10*, *IGFALS*, and *DUSP1* between Groups I and II are among the most striking detected for all hepatic transcripts (Figure 1c: orange). However, while generally listed by the PharmGKB, no clinical or variant annotations are mentioned in there on *PPP1R10* (https://www.pharmgkb.org/gene/PA33612, accessed on 7 March 2021), *IGFALS* is completely absent from this database (https://www.pharmgkb.org/gene/PA29702, accessed on 7 March 2021), and for *DUSP1*, the PharmGKB provides clinical (https://www.pharmgkb.org/clinicalAnnotation/1447961329, accessed on 7 March 2021) and variant annotations (https://www.pharmgkb.org/gene/PA27519/variantAnnotation, accessed on 7 March 2021) only for salbutamol, a β_2_-sympathomimetic asthma drug. In stark contrast, the University of Santa Cruz’s genome browser (https://genome.ucsc.edu, accessed on 7 March 2021) lists dozens of pharmaceuticals that interact with *PPP1R10*, *IGFALs*, and *DUSP1*. Both the high-level biological roles of these genes as well as their extremely high age-dependent transcription differences strongly imply that such interactions may be of utmost importance for age-appropriate drug administration and should be duly acknowledged as VIPs. In addition, we identified *ABCB1* and *ABCA9* (integral membrane transporters of the ATP-binding cassette (ABC) superfamily [58,59]) as potential pharmacogenes (Figure 3). Already listed as a VIP (https://www.pharmgkb.org/vip/PA166170352, accessed on 7 March 2021) and known to be involved in multidrug resistance [60], upregulated *ABCB1* expression in Group II now suggests its contribution to increased multidrug resistance in old age [61]. *ABCA9* transcription is induced in monocytes during MΦ differentiation and is suppressed by cholesterol import [62]. The *ABCA9* gene might therefore play a role in inflammaging, but its expression in Kupffer cells has not yet been determined.

Medawar’s mutation accumulation hypothesis suggested aging to be a consequence of the gradually declining force of natural selection [63]. Turan and colleagues confirmed its prediction that highly expressed genes in old adults are under a weaker selection pressure. They identified an age-related decrease in hepatic transcriptome conservation, which included genes for responses to age-associated tissue damage [64]. In long-lived individuals however, the expression of inflammaging- and senescence-related genes in human blood and skin cells (liver cells were not examined) was most strictly controlled when compared with mature and old individuals [65]. Consequently, an integrated view of the results of both studies suggests that, in older patients, hepatic pharmacogenes should be tested for their expression stability to arrive at individualized pharmacologic treatment recommendations.

#### 3.6.4. Transcripts Assigned to the Category ‘Miscellaneous’

The category ‘miscellaneous’ holds the genes/transcripts KIAA0040 (encoding a protein of unknown function) and γ-glutamyltransferase 5 (GGT5) (Figure 3). KIAA0040 is highly significantly associated with alcohol dependence, which let the authors conclude that this gene might harbor a causal variant for this disorder [66]. Having found a prominent young-vs.-old transcript discrepancy for KIAA0040 (Figure 2e), we thus cautiously assume that aged individuals might be less prone for developing alcohol dependence, but this conjecture must be critically scrutinized in view of numerous other genetic risk factors [67]. GGT5, which encodes a leukotriene C_4_→D_4_-converting enzyme, is expressed by hepatic Kupffer cells [68]. Presently, the lack of evidence does not justify assigning GGT5 to the ‘inflammaging’ category. However, the highly relevant difference in its expression between Groups I and II may stimulate further clarifying research in this direction.

### 3.7. Implications for Non-Alcoholic Fatty Liver Disease and Hepatocellular Carcinoma

Non-alcoholic fatty liver disease (NAFLD) with its sub-entities non-alcoholic fatty liver (NAFL) and non-alcoholic steatohepatitis (NASH) as well as hepatocellular carcinoma (HCC) are among the liver diseases with the highest global prevalences and incidences, and they are constantly on the rise [69]. However, and in stark contrast to the consequential urgent and obvious medical needs, current treatments are limited. Therefore, the prospect for any novel therapy options offering improvements in safety and efficacy is highly welcome. Our results suggest such options: As detailed hereafter, prominent age-dependent differences in the expression of especially three hepatic genes demonstrably entail an increased risk for and occurrence of liver disease that might be specifically counteracted:

(i). The CASP8- and FADD-like apoptosis regulator (*CFLAR*)-encoded protein blocks apoptosis by inhibiting procaspase-8 [70,71]. Expression of the ‘regulome’-assigned *CFLAR* gene is strongly downregulated in age (Figure 2a), which increases death receptor-mediated hepatocyte apoptosis [70,72]. Cholestatic liver injury characterized by rapid increases in intrahepatic proinflammatory parameters, hepatocyte death, hepatic stellate cell (HSC) activation, and fibrogenesis in mice exhibiting *CFLAR*^–/–^ hepatocytes [73] suggests an increased risk of cholestasis upon drastically reduced *CFLAR* expression in age. By targeting MAP3K5 kinase (thus blocking downstream signaling), CFLAR also suppresses NASH [74], which indicates that reduced *CFLAR* expression increases the likelihood for NASH development.
(ii). Expression of the ‘regulome’-assigned regulator of G-protein signaling 5 (*RGS5*) is downregulated in age. While RGS5 protects from NAFL/NASH development [75], reduced *RGS5* expression increases obesity, hepatic steatosis, inflammation, and insulin resistance [76,77]. Therefore, *RGS5* might serve as a target for NAFL/NASH-preventive approaches in progressed age. Moreover, *RGS5* downregulation induces HSC-driven liver fibrosis [78], so that therapeutic induction of *RGS5* in older individuals might also serve as an approach for tackling liver fibrosis.
(iii). Aging goes along with downregulated expression of *GATA4*, the developmental master regulator of liver sinusoidal endothelial cells (LSECs). *GATA4* deficiency in murine LSECs causes perisinusoidal liver fibrosis, hepatopathy, and impaired liver regeneration, and GATA4^+^ LSEC numbers are reduced in human cirrhotic livers. Targeting *GATA4* thus may be promising to prevent/treat liver fibrosis for reducing cirrhosis, liver failure, and/or the development of fibrosis/cirrhosis-dependent HCC [79].

Evidence thus indicates that the increase in NAFLD, liver fibrosis/cirrhosis, and HCC in progressed age [80] are promoted by downregulated *CFLAR*, *RGS5*, and/or *GATA4* expression. This understanding may offer clues for developing more potent therapeutics in the face of the insidious NAFLD pandemic and the increasing incidence of HCC [81].

### 3.8. Evidence for a Genetically Governed ‘Aging Cascade’: A Testable Hypothesis

**Figure 4:**
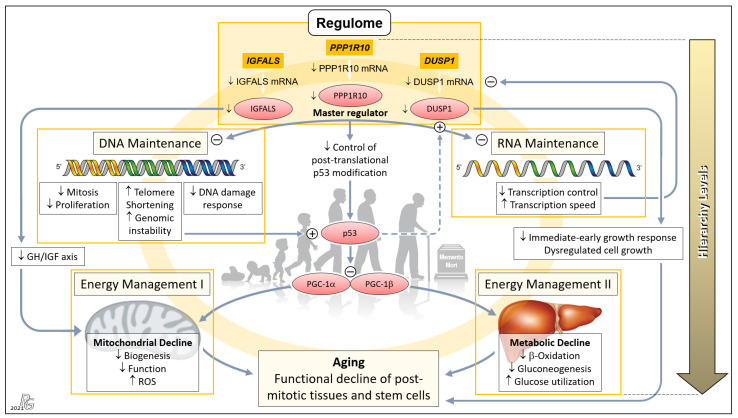
Concept of a genetically anchored regulatory ‘aging cascade’. This hierarchical top-down structure embraces the telomere–p53–PGC ‘axis of aging’ proposed earlier by Sahin and colleagues to cause the functional decline of post-mitotic tissues and stem cells [12,13]. The first hierarchical level comprises *PPP1R10*, *IGFALS*, and *DUSP1* whose transcripts were among the group of 16 mRNAs displaying the most relevant old-vs.-young differences in terms of *p* and EFS score, being led by *PPP1R10* and *DUSP1* (cf. Figure 1b). We propose that these three ‘Regulome’-assigned genes govern the process of aging. *PPP1R10* acts as the master regulator of the second hierarchical level comprising the key tasks of DNA and RNA Maintenance. Downregulation of *PPP1R10* transcription in age substantially reduces fundamental DNA-associated cellular processes and, conversely, increases telomere shortening and genomic instability. The latter were the top aspects of the earlier proposed ‘axis of aging’, leading to induction of p53 and the resultant repression of PPARγ coactivators 1α and 1β (PGC-1α; PGC-1β), which detrimentally affect the third hierarchical level comprising Energy Management I and II. Downregulation of the *PPP1R10* transcript also negatively impinges upon RNA Maintenance at the second hierarchical level, which, in a negative feedback loop, further impairs the transcription of *PPP1R10*, *IGFALS*, and *DUSP1* (as well as, obviously, other transcripts in progressed age). Next, downregulation of *IGFALS* transcription/translation, via impairing the essential growth hormone/insulin-like growth factor (GH/IGF) axis, causes substantial mitochondrial decline and aging. Finally, downregulated *DUSP1* transcription/translation acts to the detriment of the immediate-early growth response and causes dysregulated cell growth, which entails serious cellular impairment and aging. Besides belonging to the ‘regulome’ category, *PPP1R10* and *IGFALS* intersect with the ‘regeneration’ and ‘inflammaging” categories (Figure 3), which further enhances their impact upon downregulated expression in age. The proposed ‘aging cascade’ is visualized by a light orange ring connecting the three hierarchical levels and the consequence of aging. (Abbreviation: ROS, reactive oxygen species.) Background image: clipartstation.com (modified).

Based on the role of the liver as an organ of major systemic relevance; highly significant age-related differences in the expression of *PPP1R10*, *IGFALS*, and *DUSP1*; and ample evidence on the action of their protein products on central aging-associated downstream processes, we now present a broadly substantiated hypothesis on a *PPP1R10/IGFALS/DUSP1*-governed ‘aging cascade’. Using a mouse model of aging, Sahin et al. earlier elegantly combined progressive DNA damage and mitochondrial decline via p53 as an ‘axis of aging’. This axis implicates a chain reaction leading from telomere dysfunction to the induction of p53, and the resultant repression of PPARγ coactivators 1α and 1β, which detrimentally affects key cellular mechanisms, hence propelling the process of aging [12]. Our hierarchically arranged hypothesis embraces and extends this model: Therein, three ‘regulome’-assigned genes assume paramount positions (Figure 4).

The *PPP1R10* transcript displayed by far the most significant and striking age-related difference among all protein-encoding hepatic transcripts (Figure 1c and Figure 2a). This difference, together with the essential functions of *PPP1R10*, clearly underscores its dominance as an ontogenetic master regulator. The *PPP1R10*-encoded enzyme harnesses the protein kinase A-regulated serine/threonine protein phosphatase 1 (PP1) to establish a PPP1R10:PP1 holoenzyme [82] that comprehensively acts on the upstream DNA and RNA levels and—via the established ‘axis of aging’—the downstream levels of energy management (Figure 4). At the DNA/RNA levels, this gene acts as the master regulator of mitosis/proliferation/apoptosis [24,25,26,27], influences the DNA damage response [83,84], controls RNA processing [25,83], and negatively regulates the RNA transcription speed [85]. Dwindling transcription in aging thus lets *PPP1R10* gradually lose its grip on these fundamental processes. Element #1 of the ‘axis of aging’—telomere shortening/genomic instability—results from reduced nuclear concentrations of PPP1R10:PP1 [86,87,88]. In aging, the loss of control by *PPP1R10* over RNA processing and transcription speed creates a negative feedback loop on the transcription of *PPP1R10*, *IGAFLS*, and *DUSP1*, which further accelerates the aging process. Next, PPP1R10 usually plays an important role in controlling cell death in response to cellular stresses by post-translational p53 modification [89]. Thus, p53—i.e., element #2 of the ‘axis of aging’—escapes *PPP1R10* control once its expression drops. Moreover, the induction and regulation of *DUSP1* transcription by p53 [90,91] is further undermined by the impaired *PPP1R10*-dependent RNA maintenance in age (Figure 4: Dashed arrow).

Hierarchically subordinate to *PPP1R10*, *IGFALS*, and *DUSP1* (cf. Figure 1c and Figure 2a) act on the downstream aspects of aging only. First, the gene for insulin-like growth factor-binding protein acid-labile subunit (*IGFALS*) significantly contributes to an individual’s lifetime. As an essential component of the GH/IGF axis with its proven role in longevity, it prolongs the half-life of insulin-like growth factor (IGF)-I and IGF-II [92]. In contrast, serious developmental harm or disease result from *IGFALS* defects [93,94,95], mutations [96], or IGFALS protein deficiency [97,98]. The same axis regulates mitochondrial processes whose decline upon reduced IGFALS concentrations are hallmarks of aging [99] (Figure 4). Second, downregulated transcription of the dual specificity phosphatase 1 (*DUSP1*) gene impairs the immediate-early growth response and entails dysregulated cell growth [28,29], which fundamentally weakens the body’s regenerative capacities. Concerted *PPP1R10/IGFALS/ DUSP1* action funnels into the aging-associated functional decline of post-mitotic tissues and stem cells. Therefore, a hierarchically arranged network emerges whose main components connect as an ‘aging cascade’ (Figure 4).

**Figure 5:**
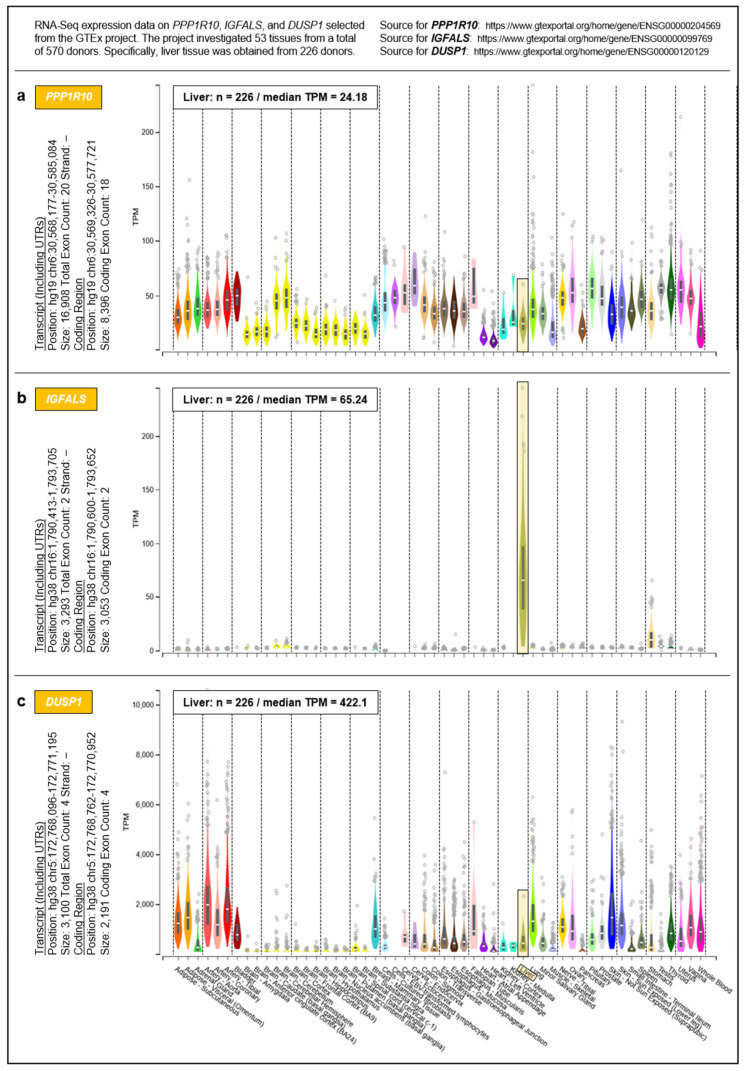
Systemic expressions of (**a**) *PPP1R10*, (**b**) *IGFALS*, and (**c**) *DUSP1*. Expressions were determined by the GTEx project in a total of 53 tissues. For inter-tissue comparison, the expressions of *PPP1R10*, *IGFALS*, and *DUSP1* in the liver are highlighted.

To address the question of whether the proposed governance by *PPP1R10*, *IGFALS*, and *DUSP1* can be generalized from the hepatic transcriptome to an entire organism, we selected respective data from the Genotype-Tissue Expression (GTEx) project obtained from 53 tissues of 570 donors (Figure 5). For a complete overview of the GTEx project’s tissue and sample statistics, we refer to the respective condensed information provided on its portal: https://www.gtexportal.org/home/tissueSummaryPage (accessed on 7 March 2021). According to the GTEx data, (i) the expression of *PPP1R10* as a global master regulator is ubiquitous and tightly regulated; (ii) the expression of *IGFALS* is essentially confined to the liver, but by serving as a transporter for IGF-I/IGF-II its protein product is crucial for the functionality of the systemic GH/IGF axis whereby assuming a key role in steering an individual’s development and lifespan; and (iii) the expression of *DUSP1* as a gene implicated in immediate-early and overall cell growth is regulated locally depending on a tissues’ specific growth requirements. Therefore, the findings/ interpretations for the liver transcriptome on these genes can, at least in principle, be generalized to the individual. All data, supportive information, and derivations thus justify postulating a genetically anchored regulatory ‘aging cascade’.

If confirmed experimentally, further research will, inter alia, need to clarify the following:

(i). The potential existence and role of ethnic differences;
(ii). Whether the expressions of the individually specific MHC I proteins and PPP1R10 are jointly regulated, as PPP1R10 is intriguingly encoded on chromosome 6 within the MHC I region: https://www.pharmgkb.org/gene/PA33612 (accessed on 7 March 2021);
(iii). In which way PPP1R10 is interconnected with the organism’s central clock located in the suprachiasmatic nucleus and its regulation of melatonin secretion (with resultant pro- and anti-inflammatory effects, among others) [100,101] as well as the recently identified inflammatory aging clock [102], and with the age-dependent reprogramming of the circadian transcriptome discovered in the murine liver [103]; and
(iv). Especially from a clinician’s point of view, whether pharmaceuticals modifying the transcription of PPP1R10, IGFALS, and DUSP1 (Table S5) may affect an individual’s lifetime.

In addition, potential modulatory effects of age-dependent differences in other RNA species—e.g., the long non-coding RNAs (lncRNAs) (cf. Table 1)—still largely remain to be analyzed in humans. Indeed, results by White and colleagues in the aging mouse liver point in exactly this direction by showing upregulation of distinct lncRNAs in the aging liver whereby suggesting a putative regulatory hotspot locus in aging liver, as well as discrete roles of certain lncRNAs within interaction networks that, for instance, are implicated in inflammaging and regeneration [104].

If key issues such as these can be resolved, one might theoretically envision measures to counteract the gradual loss in *PPP1R10* expression, which is supposed to chiefly time an individual’s biological decline. However, interfering with this presumptive ontogenetic master regulator would promise to be a most critical and potentially highly perilous undertaking, and it will inevitably be burdened by profound ethical considerations.

## Figures and Tables

**Table 1:** Biotypes ^1^ of initially detected transcripts. ^2^ (Abbreviation: TPM, transcripts per million.).

Biotype	Number of Transcripts				
Filter	None	Mean TPM > 1	Single TPM > 50	*p* < 0.05	Ratio > 1.4
None: All genes	60,617	15,470	3922	263	95
Protein-encoding	19,986	12,930	3818	257	89
Pseudogenes	22	-	-	-	-
Processed pseudogenes	10,170	318	7	-	-
Unprocessed pseudogenes	2626	62	4	-	-
Polymorphic pseudogenes	42	5	4	-	-
Unitary pseudogenes	97	2	-	-	-
Transcribed processed pseudogenes	491	48	-	-	-
Transcribed unprocessed pseudogenes	916	173	11	1	1
Transcribed unitary pseudogenes	129	23	-	-	-
Translated processed pseudogenes	2	-	-	-	-
Translated unprocessed pseudogenes	2	-	-	-	-
rRNA pseudogenes	499	3	-	-	-
IG pseudogene	1	-	-	-	-
IG C pseudogenes	9	2	-	-	-
IG J pseudogenes	3	-	-	-	-
IG V pseudogenes	188	-	-	-	-
TR V pseudogenes	33	-	-	-	-
TR J pseudogenes	4	-	-	-	-
Mt rRNAs	2	2	2	-	-
Mt tRNAs	22	1	-	-	-
miRNAs	1879	3	-	-	-
Misc RNAs	2220	12	-	-	-
rRNAs	58	-	-	-	-
scRNA	1	-	-	-	-
snRNAs	1910	8	-	-	-
snoRNAs	942	11	-	-	-
Ribozymes	8	-	-	-	-
sRNAs	5	-	-	-	-
scaRNAs	49	-	-	-	-
vaultRNA	1	-	-	-	-
IG C genes	14	12	10	-	-
IG D genes	37	-	-	-	-
IG J genes	18	-	-	-	-
IG V genes	144	45	3	-	-
TR C genes	6	4	-	-	-
TR D genes	4	-	-	-	-
TR J genes	79	-	-	-	-
TR V genes	106	1	-	-	-
lncRNAs	16,828	1693	63	5	5
TEC ^3^	1064	112	-	-	-

^1^ A biotype is defined as the genotype shared or its distinguishing peculiarity. ^2^ Sources: 1. https://www.gencodegenes.org/pages/biotypes.html (accessed on 7 March 2021); 2. https://vega.archive.ensembl.org/info/about/gene_and_transcript_types.html (accessed on 7 March 2021). ^3^ TEC: To be experimentally confirmed [category used for non-spliced expressed sequence tag (EST) clusters with polyA features originally created for the ENCODE Project [4,6] to highlight regions that could indicate the presence of protein-encoding genes that require experimental validation by 5′ RACE or RT-PCR to extend the transcripts, or by confirming expression of the putatively encoded peptides with specific antibodies].

**Table 2:** Confirmation of next-generation sequencing (NGS) results of select transcripts of relevance by qRT-PCR. Hepatic transcript expressions are listed in the order of relevance. The transcript ratios obtained by NGS were generally confirmed by fold-change via qRT-PCR. Only the transcripts of the genes *FAH* and *LIPC* revealed contrary results. (Abbreviations: CV, coefficient of variation; Cp, crossing point; Grp., Group).

	qRT-PCR	NGS				
Gene ID ^1^	Fold Change	Ratio	Mean Cp	Mean Cp	% CV	% CV
		$\frac{Grp.\;I}{Grp.\;II}$	Grp. I	Grp. II	Grp. I	Grp. II
*PPP1R10*	1.701	1.580	34.90	34.24	2.98	1.78
*DUSP1*	3.095	1.762	26.00	26.21	5.14	3.55
*HSD17B14*	8.508	3.854	30.13	31.80	3.06	3.56
*FAH*	1.175	0.668	27.55	26.37	2.79	1.28
*PALLD*	1.116	1.719	37.73	36.47	3.94	0.95
*AGO2*	1.376	1.496	38.55	37.85	2.50	3.20
*TFF3*	94.403	63.814	31.46	36.86	14.87	4.13
*KIAA0040*	2.680	1.842	34.24	34.51	4.24	2.86
*CFLAR*	1.803	1.427	26.37	25.91	3.25	1.74
*ITSN1*	3.151	1.427	37.20	37.55	3.54	4.90
*FLNA*	1.768	1.692	33.17	32.84	2.55	1.60
*LIPC*	1.585	0.686	25.53	25.03	2.08	1.85
*IGFALS*	2.707	1.977	33.65	33.78	3.68	3.68
*CYP3A43*	3.213	2.473	25.78	26.16	4.14	3.09
*GATA4*	2.980	3.405	32.38	32.64	4.59	2.65
*EGR1*	8.313	2.121	31.26	33.01	7.46	12.18

^1^ Gene acronyms: *AGO2*, argonaute RISC catalytic component 2; *CFLAR*, CASP8- and FADD-like apoptosis regulator; *CYP3A43*, cytochrome P450 family 3 subfamily A member 43; *DUSP1*, dual-specificity phosphatase 1; *EGR1*, early growth response 1; *FAH*, fumarylacetoacetate hydrolase; *FLNA*, filamin A; *GATA4*, GATA-binding protein 4; *HSD17B14*, hydroxysteroid 17-β dehydrogenase 14; *IGFALS*, insulin-like growth factor-binding protein acid-labile subunit; *ITSN1*, intersectin 1; *KIAA0040*, uncharacterized protein KIAA0040; *LIPC*, lipase C, hepatic type; *PALLD*, palladin, cytoskeletal associated protein; *PPP1R10*, protein phosphatase 1 regulatory subunit 10; *TFF3*, trefoil factor 3.

## Data Availability

All raw and processed sequencing data generated in this study have been submitted to the NCBI Gene Expression Omnibus (GEO; https://www.ncbi.nlm.nih.gov/geo/, accessed on 7 March 2021). The datasets and the computer code produced in this study are available under: https://www.ncbi.nlm.nih.gov/geo/query/acc.cgi?acc=GSE183915 (accessed on 7 March 2021).

## Supplementary Materials

The following are available online at https://www.mdpi.com/article/10.3390/pharmaceutics13122009/s1, Table S1. Patients’ age and gender in order of increasing age. (Abbreviations: m, male; f, female.), Table S2. Human target transcripts for qRT-PCR. The list starts with the reference gene, followed by genes of the transcripts investigated in alphabetical order, Table S3. Gene expressions of interest identified by applying EFS software. Transcripts are listed from most relevant to least relevant according to their EFS scores (>0.3: 16 transcripts/>0.2: 28 further transcripts). For full names of protein products, cf. Table 2. Increases in EFS scores indicate increasing relevance when comparing young and old patients. Ratios between Group (Grp.) I and Group II differing by a factor of at least × 2 (i.e., ≤0.5 or ≥2.0) are boxed (red data in Figure 2), while ratios between >0.5 and <2.0 are underlined (blue data in Figure 2). Data for all 44 transcripts are at least statistically significant (*p* < 0.05). (Abbreviations: Grp., Group; TPM, transcripts per million.), Table S4. Specifications for gene transcripts identified by EFS software. Genes related to the 44 hepatic transcripts matching the EFS score cutoff of >0.2 are tabulated according to their chromosome (Chr) location, wherein listed alphabetically according their gene designations. Especially high ortholog/paralog^1^ ratios (with ≤9 paralogs) are boxed. As summarized in the bottom section, gene transcripts of relevance were expressed from all human chromosomes, except for Chr 18 and Chr Y. Hence, no statistically significant age-dependent transcript differences were attributable to the male sex chromosome. Transcript numbers (##) with relevant age-dependent differences were six for Chr 1; four for Chr 22; three for Chr 3, 5, and 11; two for Chr 2, 6, 10, 12, 15, 16, 19, and X; and one for Chr 4, 7, 8, 9, 13, 14, 17, 20, and 21, respectively. Information was compiled from https://www.ensembl.org/Homo_sapiens/Info/Index (accessed on 16 April 2021), Table S5. Pharmaceuticals modifying the mRNA expression of select regulome-assigned hepatic genes that display highly significant age-dependent differences in expression. Active pharmaceutical ingredients (APIs) that affect (A), decrease (↓), or increase (↑) the transcription of *PPP1R10*, *IGFALS*, or *DUSP1* are listed in alphabetical order. The term, ‘affects’, indicates that the respective studies referred to in the Comparative Toxicogenomics Database generated ambiguous results. APIs interfering with the expression of ≥2 of the three regulome-assigned genes are boxed.
