# Supplementary material for: Transcriptome-Wide Analysis of Human Liver Reveals Age-Related Differences in the Expression of Select Functional Gene Clusters and Evidence for a PPP1R10-Governed ‘Aging Cascade’
Source: Pharmaceutics. 2021 Nov 25;13(12):2009. doi: 10.3390/pharmaceutics13122009 (PMC8709089; doi:10.3390/pharmaceutics13122009)
Supplement: Supplementary file 1 [file pharmaceutics-13-02009-s001.zip › pharmaceutics-1455184-supplementary.pdf]

pharmaceutics

MDPI

# Supplementary Materials: Transcriptome-Wide Analysis of Human Liver Reveals Age-Related Differences in the Expression of Select Functional Gene Clusters and Evidence for a *PPP1R10*-Governed 'Aging Cascade'

**Thomas Schreiter, Robert K. Gieseler, Ramiro Vílchez-Vargas, Ruy Jauregui, Jan-Peter Sowa, Susanne Klein-Scory, Ruth Broering, Roland S. Croner, Jürgen W. Treckmann, Alexander Link and Ali Canbay**

**Table S1.** Demographic and clinical characteristics of patient groups I and II in order of gender and increasing age. Prior to surgery, all patients tested negative for the hepatitis viruses HAV, HBV, HCV, and HDV as well as HIV-1. All patients underwent surgery before the onset of the COVID-19 pandemic and, thus, were not tested for SARS-CoV-2. None of the patients had received radiotherapy. (Abbreviations: AHT, arterial hypertension; BEP, bleomycin + etoposide + cisplatin; CA, carcinoma; CCA, cholangiocellular carcinoma; CP/PT, carboplatin + paclitaxel; f, female; FOLFOX, folinic acid + fluorouracil + oxaliplatin; FNH, focal nodular hyperplasia; HCC, hepatocellular carcinoma; HCA, hepatocellular adenoma; HHEx, hemihepatectomy; m, male; mets, metastases; NASH, non-alcoholic steatohepatitis; pre-OP, preoperatively; RES, resection; SEG, segment or segment; T1DM or T2DM, type 1 or type 2 diabetes mellitus; TC, testicular cancer.).

| Group I: Young | | | | | | | | | |
|---|---|---|---|---|---|---|---|---|---|
| Demographic Information | | | | Medication | Comorbidities or Additional Information | | | | |
| Gender | Age | Underlying Disease | Surgical Intervention | Preoperative Chemotherapy | Diabetes | Steatosis (Grade) | NASH | Fibrosis (Grade) | Other |
| f | 23 | HCA | Right HHEx | – | – | – | – | – | Bronchial asthma |
| f | 26 | FNH | Right HHEx + Atypical RES: SEG I, II, III | – | – | – | – | – | – |
| f | 26 | FNH | RES: SEG II, III, VII | – | – | 2 | Yes | – | – |
| f | 34 | FNH | Typical RES: SEG IV | – | – | – | – | – | Obesity |
| f | 43 | Cervical CA: Mets | Left HHEx | CP/PT | T1DM | – | – | – | – |
| f | 47 | CCA | Right HHEx | – | – | – | – | – | Substituted hypothyreosis after hemithyroidectomy 3 yrs. pre-OP |
| m | 25 | TC: Mets | RES: SEG V, VI + atypical RES: SEG III | BEP | – | – | – | – | – |
| m | 46 | HCA | RES: SEG II, III | – | T2DM | 2 | Yes | – | AHT |
| m | 48 | Colon CA: Mets | RES: SEG II, III, IVa, VI, VIII | FOLFOX | – | – | – | – | – |

| Group II: Old | | | | | | | | | |
|---|---|---|---|---|---|---|---|---|---|
| Demographic Information | | | | Medication | Comorbidities or Additional Information | | | | |
| Gender | Age | Underlying Disease | Surgical Intervention | Preoperative Chemotherapy | Diabetes | Steatosis (Grade) | NASH | Fibrosis (Grade) | Other |

| | | | | | | | | | |
|---|---|---|---|---|---|---|---|---|---|
| f | 79 | Colon CA: Mets | Right HHEx | – | – | – | – | – | Multinodular goiter |
| f | 79 | CCA | Right HHEx | – | – | 2 | – | F1 | AHT |
| m | 75 | HCC | RES: SEG VI | – | – | 1 | – | F4 | AHT |
| m | 76 | Colon CA: Mets | Right HHEx + atypical RES: SEG II | – | T2DM | – | – | – | AHT |
| m | 76 | Sigma CA: Mets | RES: SEG VIb, V | – | – | – | – | F0–F1 | AHT |
| m | 78 | Colon CA: Mets | Right HHEx | – | – | 2 | – | F2 | AHT |
| m | 78 | CCA | RES: SEG II, III | – | – | – | – | F1 | AHT, obesity |
| m | 78 | CCA | Extended right HHEx | – | – | – | – | – | In-situ split 2 wks. pre-OP |

**Table S2.** Human target transcripts for qRT-PCR. The list starts with the reference gene, followed by genes of the transcripts investigated in alphabetical order.

| **Genes**[1] | **Sequences** | **Genes**[1] | **Sequences** |
|---|---|---|---|
| *PPIA* (reference) | | | |
| Main target | ENST00000468812 | | |
| Amplicon length | 114 | | |
| Number of targets | 4 | | |
| *CFLAR* | | *HSD17B14* | |
| Main target | ENST00000309955 | Main target | ENST00000596349 |
| Amplicon length | 109 | Amplicon length | 86 |
| Number of targets | 7 | Number of targets | 4 |
| *DUSP1* | | *IGFALS* | |
| Main target | ENST00000239223 | Main target | ENST00000415638 |
| Amplicon length | 116 | Amplicon length | 67 |
| Number of targets | 1 | Number of targets | 1 |
| *EGR1* | | *KIAA0040* | |
| Main target | ENST00000239938 | Main target | ENST00000563563 |
| Amplicon length | 127 | Amplicon length | 131 |
| Number of targets | 1 | Number of targets | 6 |
| *FAH* | | *LIPC* | |
| Main target | ENST00000537726 | Main target | ENST00000560664 |
| Amplicon length | 117 | Amplicon length | 84 |
| Number of targets | 10 | Number of targets | 6 |
| *FLNA* | | *PPP1R10* | |
| Main target | ENST00000420627 | Main target | ENST00000376511 |
| Amplicon length | 141 | Amplicon length | 103 |
| Number of targets | 7 | Number of targets | 2 |
| *GATA4* | | *TFF3* | |
| Main target | ENST00000526716 | Main target | ENST00000398431 |
| Amplicon length | 81 | Amplicon length | 68 |
| Number of targets | 1 | Number of targets | 4 |

[1] *CFLAR*, CASP8- and FADD-like apoptosis regulator; *DUSP1*, dual specificity phosphatase 1; *EGR1*, early growth response 1; *FAH*, fumarylacetoacetate hydrolase; *FLNA*, filamin A; *GATA4*, GATA-binding protein 4; *HSD17B14*, hydroxysteroid 17-β dehydrogenase 14; *IGFALS*, insulin-like growth factor-binding protein acid labile subunit; *KIAA0040*, uncharacterized protein KIAA0040; *LIPC*, lipase C, hepatic type; *PPP1R10*, protein phosphatase 1 regulatory subunit 10; *PPIA*, peptidyl-prolyl *cis-trans* isomerase A; *TFF3*, trefoil factor 3.

**Table S3.** Gene expressions of interest identified by applying EFS software. Transcripts are listed from most relevant to least relevant according to their EFS scores (>0.3: 16 transcripts / >0.2: 28 further transcripts). For full names of protein products, *cf.* Table 2. Increases in EFS scores indicate increasing relevance when comparing young and old patients. Ratios between Group (Grp.) I and Group II differing by a factor of at least ×2 (i.e., ≤0.5 or ≥2.0) are boxed (red data in Figure 2), while ratios between >0.5 and <2.0 are underlined (blue data in Figure 2). Data for all 44 transcripts are at least statistically significant ($p$ <0.05). (Abbreviations: Grp., Group; TPM, transcripts per million.).

| Gene ID | Gene | Transcription Rates [TPM] Maximum | Mean Grp. I | Mean Grp. II | Ratio Grp. II / Grp. I | $p$ (Two-tailed) | | EFS Score |
|---|---|---|---|---|---|---|---|---|
| ENSG00000204569 | *PPP1R10* | 159.20 | 107.86 | 67.79 | 0.629 | 0.0003 | > 0.3 | 0.61415 |
| ENSG00000120129 | *DUSP1* | 557.40 | 375.44 | 212.30 | 0.565 | 0.0055 | | 0.55957 |
| ENSG00000087076 | *HSD17B14* | 90.77 | 47.32 | 12.74 | 0.269 | 0.0010 | | 0.53103 |
| ENSG00000148120 | *AOPEP* | 179.15 | 114.07 | 66.16 | 0.580 | 0.0010 | | 0.40353 |
| ENSG00000185745 | *IFIT1* | 70.04 | 23.61 | 41.94 | 1.776 | 0.0360 | | 0.36984 |
| ENSG00000235750 | *KIAA0040* | 61.92 | 27.85 | 14.67 | 0.527 | 0.0016 | | 0.36721 |
| ENSG00000196924 | *FLNA* | 92.67 | 56.39 | 33.07 | 0.586 | 0.0016 | | 0.35981 |
| ENSG00000003402 | *CFLAR* | 123.14 | 93.58 | 65.16 | 0.696 | 0.0006 | | 0.33686 |
| ENSG00000085563 | *ABCB1* | 54.81 | 29.32 | 42.30 | 1.442 | 0.0206 | | 0.33654 |
| ENSG00000103876 | *FAH* | 301.97 | 166.06 | 250.17 | 1.506 | 0.0111 | | 0.32981 |
| ENSG00000153250 | *RBMS1* | 52.92 | 38.16 | 25.51 | 0.668 | 0.0152 | | 0.32777 |
| ENSG00000099769 | *IGFALS* | 471.76 | 261.59 | 126.81 | 0.485 | 0.0037 | | 0.32758 |
| ENSG00000117616 | *RSRP1* | 162.29 | 108.48 | 73.63 | 0.679 | 0.0274 | | 0.32683 |
| ENSG00000089127 | *AC004551.1* | 58.72 | 21.41 | 32.94 | 1.538 | 0.0206 | | 0.32390 |
| ENSG00000144642 | *RBMS3* | 85.14 | 44.54 | 29.68 | 0.666 | 0.0055 | | 0.31905 |
| ENSG00000160180 | *TFF3* | 397.12 | 81.92 | 1.25 | 0.015 | 0.0055 | | 0.31574 |
| ENSG00000126012 | *KDM5C* | 51.25 | 30.87 | 19.87 | 0.643 | 0.0111 | > 0.2 | 0.28884 |
| ENSG00000152818 | *UTRN* | 207.84 | 125.80 | 70.80 | 0.563 | 0.0010 | | 0.28580 |
| ENSG00000101335 | *MYL9* | 64.72 | 41.33 | 22.73 | 0.550 | 0.0079 | | 0.26749 |
| ENSG00000183963 | *SMTN* | 65.16 | 36.07 | 21.85 | 0.606 | 0.0152 | | 0.26602 |
| ENSG00000143248 | *RGS5* | 337.12 | 131.96 | 60.40 | 0.458 | 0.0152 | | 0.25855 |
| ENSG00000074276 | *CDHR2* | 73.29 | 20.56 | 47.55 | 2.313 | 0.0111 | | 0.25525 |
| ENSG00000100234 | *TIMP3* | 276.12 | 168.16 | 99.01 | 0.589 | 0.0206 | | 0.25389 |
| ENSG00000166035 | *LIPC* | 163.41 | 85.65 | 124.67 | 1.456 | 0.0360 | | 0.24764 |
| ENSG00000111077 | *TNS2* | 118.89 | 78.25 | 52.68 | 0.673 | 0.0206 | | 0.24403 |
| ENSG00000105676 | *ARMC6* | 113.94 | 78.93 | 51.58 | 0.654 | 0.0464 | | 0.23935 |
| ENSG00000075240 | *GRAMD4* | 167.99 | 62.66 | 21.86 | 0.349 | 0.0274 | | 0.23830 |
| ENSG00000132254 | *ARFIP2* | 60.99 | 33.71 | 22.60 | 0.670 | 0.0206 | | 0.23406 |
| ENSG00000184226 | *PCDH9* | 104.44 | 43.80 | 21.61 | 0.493 | 0.0274 | | 0.23297 |
| ENSG00000099998 | *GGT5* | 92.41 | 42.52 | 27.77 | 0.653 | 0.0464 | | 0.22929 |
| ENSG00000010322 | *NISCH* | 61.59 | 37.57 | 22.94 | 0.611 | 0.0206 | | 0.22850 |
| ENSG00000120738 | *EGR1* | 727.96 | 381.72 | 174.46 | 0.457 | 0.0360 | | 0.22582 |

| | | | | | | | |
|---|---|---|---|---|---|---|---|
| ENSG00000149541 | *B3GAT3* | 54.18 | 22.17 | 15.52 | 0.700 | 0.0464 | 0.22514 |
| ENSG00000136574 | *GATA4* | 120.06 | 44.12 | 12.75 | 0.289 | 0.0274 | 0.22168 |
| ENSG00000154258 | *ABCA9* | 64.27 | 37.59 | 24.27 | 0.646 | 0.0274 | 0.22112 |
| ENSG00000054282 | *SDCCAG8* | 66.80 | 45.26 | 31.39 | 0.693 | 0.0037 | 0.22020 |
| ENSG00000054654 | *SYNE2* | 229.66 | 146.72 | 94.88 | 0.647 | 0.0274 | 0.21864 |
| ENSG00000257017 | *HP* | 52678.43 | 16805.05 | 29002.98 | 1.726 | 0.0360 | 0.21811 |
| ENSG00000163660 | *CCNL1* | 249.51 | 185.39 | 131.54 | 0.710 | 0.0274 | 0.21788 |
| ENSG00000058668 | *ATP2B4* | 85.89 | 40.48 | 27.22 | 0.673 | 0.0464 | 0.21358 |
| ENSG00000107796 | *ACTA2* | 82.20 | 51.34 | 21.13 | 0.411 | 0.0055 | 0.21218 |
| ENSG00000156234 | *CXCL13* | 78.00 | 1.79 | 14.03 | 7.858 | 0.0360 | 0.20827 |
| ENSG00000117318 | *ID3* | 113.79 | 74.94 | 45.82 | 0.611 | 0.0111 | 0.20143 |
| ENSG00000110719 | *TCIRG1* | 88.64 | 57.28 | 39.21 | 0.684 | 0.0206 | 0.20042 |

**Table S4.** Specifications for gene transcripts identified by EFS software. Genes related to the 44 hepatic transcripts matching the EFS score cutoff of >0.2 are tabulated according to their chromosome (Chr) location, wherein listed alphabetically according their gene designations. Especially high ortholog/paralog[1] ratios (with ≤9 paralogs) are boxed. As summarized in the bottom section, gene transcripts of relevance were expressed from all human chromosomes, except for Chr 18 and Chr Y. Hence, no statistically significant age-dependent transcript differences were attributable to the male sex chromosome. Transcript numbers (##) with relevant age-dependent differences were six for Chr 1; four for Chr 22; three for Chr 3, 5, and 11; two for Chr 2, 6, 10, 12, 15, 16, 19, and X; and one for Chr 4, 7, 8, 9, 13, 14, 17, 20, and 21, respectively. Information was compiled from https://www.ensembl.org/Homo_sapiens/Info/Index (accessed on 2021-04-16).

| Gene ID | Gene | Full Name of Protein Product (Comments) | Location | Gene Information | | | |
|---|---|---|---|---|---|---|---|
| | | | | Transcripts | Alleles | Orthologs[1] | Paralogs[1] |
| ENSG00000058668 | *ATP2B4* | ATPase plasma membrane $Ca^{2+}$-transporting 4 | Chr 1 | 7 | 0 | 221 | 22 |
| ENSG00000117318 | *ID3* | Inhibitor of DNA binding 3 (a so-called 'bHLH protein')[2] | | 3 | 1 | 201 | 3 |
| ENSG00000235750 | *KIAA0040* | KIAA0040 (uncharacterized) | | 6 | 0 | 5 | 0 |
| ENSG00000143248 | *RGS5* | Regulator of G protein signaling 5 | | 12 | 0 | 202 | 23 |
| ENSG00000117616 | *RSRP1* | Arginine- and serine-rich protein 1 | | 22 | 0 | 114 | 9 |
| ENSG00000054282 | *SDCCAG8* | SHH signaling and ciliogenesis regulator SDCCAG8 | | 11 | 1 | 90 | 0 |
| ENSG00000003402 | *CFLAR* | CASP8- and FADD-like apoptosis regulator | Chr 2 | 25 | 0 | 222 | 15 |
| ENSG00000153250 | *RBMS1* | RNA-binding motif single-stranded interacting protein 1 | | 15 | 0 | 265 | 24 |
| ENSG00000163660 | *CCNL1* | Cyclin L1 | Chr 3 | 26 | 0 | 213 | 6 |
| ENSG00000010322 | *NISCH* | Nischarin | | 14 | 0 | 205 | 1 |
| ENSG00000144642 | *RBMS3* | RNA-binding motif single-stranded interacting protein 3 | | 20 | 0 | 261 | 24 |
| ENSG00000156234 | *CXCL13* | C-X-C motif chemokine ligand 13 | Chr 4 | 2 | 0 | 111 | 12 |
| ENSG00000074276 | *CDHR2* | Cadherin-related family member 2 | Chr 5 | 7 | 0 | 118 | 34 |
| ENSG00000120129 | *DUSP1* | Dual specificity phosphatase 1 | | 1 | 0 | 204 | 30 |
| ENSG00000120738 | *EGR1* | Early growth response 1 | | 1 | 0 | 225 | 4 |
| ENSG00000204569 | *PPP1R10* | Protein phosphatase 1 regulatory subunit 10 | Chr 6 | 7 | 1 | 197 | 2 |
| ENSG00000152818 | *UTRN* | Utrophin | | 13 | 0 | 261 | 36 |
| ENSG00000085563 | *ABCB1* | ATP-binding cassette subfamily B member 1 | Chr 7 | 11 | 0 | 159 | 11 |
| ENSG00000136574 | *GATA4* | GATA-binding protein 4 | Chr 8 | 9 | 1 | 206 | 7 |
| ENSG00000148120 | *AOPEP* | Aminopeptidase O (putative) | Chr 9 | 21 | 0 | 224 | 22 |
| ENSG00000107796 | *ACTA2* | Actin $\alpha_2$ (smooth muscle) | Chr 10 | 6 | 0 | 149 | 27 |
| ENSG00000185745 | *IFIT1* | Interferon-induced protein with tetratricopeptide repeats 1 | | 2 | 0 | 507 | 4 |
| ENSG00000149541 | *B3GAT3* | β-1,3-Glucuronyltransferase 3 | Chr 11 | 6 | 0 | 194 | 2 |

| | | | | | | | |
|---|---|---|---|---|---|---|---|
| ENSG00000132254 | *ARFIP2* | ADP ribosylation factor interacting protein 2 | | 10 | 0 | 266 | 2 |
| ENSG00000110719 | *TCIRG1* | T-cell immune regulator 1, ATPase $H^+$-transporting V0 subunit $a_3$ (a.k.a. V-type proton ATPase 116 kDa subunit $a_3$) | | 16 | 0 | 233 | 3 |
| ENSG00000089127 | *AC004551.1* | 2'-5'-Oligoadenylate synthetase 1 | Chr 12 | 33 | 0 | 148 | 3 |
| ENSG00000111077 | *TNS2* | Tensin 2 | | 20 | 0 | 115 | 1 |
| ENSG00000184226 | *PCDH9* | Protocadherin 9 | Chr 13 | 6 | 0 | 220 | 34 |
| ENSG00000054654 | *SYNE2* | Spectrin repeat-containing nuclear envelope protein 2 | Chr 14 | 31 | 0 | 308 | 36 |
| ENSG00000103876 | *FAH* | Fumarylacetoacetate hydrolase | Chr 15 | 14 | 0 | 204 | 3 |
| ENSG00000166035 | *LIPC* | Lipase C (hepatic type) | | 7 | 0 | 228 | 9 |
| ENSG00000257017 | *HP* | Haptoglobin | Chr 16 | 18 | 0 | 137 | 17 |
| ENSG00000099769 | *IGFALS* | Insulin-like growth factor-binding protein acid labile subunit | | 3 | 0 | 187 | 22 |
| ENSG00000154258 | *ABCA9* | ATP-binding cassette subfamily A member 9 | Chr 17 | 8 | 0 | 105 | 11 |
| – | – | – | Chr 18 | – | – | – | – |
| ENSG00000105676 | *ARMC6* | Armadillo repeat containing 6 | Chr 19 | 20 | 0 | 201 | 0 |
| ENSG00000087076 | *HSD17B14* | Hydroxysteroid 17-β-dehydrogenase 14 | | 4 | 0 | 162 | 26 |
| ENSG00000101335 | *MYL9* | Myosin light chain 9 | Chr 20 | 2 | 0 | 229 | 7 |
| ENSG00000160180 | *TFF3* | Trefoil factor 3 | Chr 21 | 3 | 0 | 0 | 0 |
| ENSG00000099998 | *GGT5* | γ-Glutamyltransferase 5 | Chr 22 | 6 | 0 | 342 | 7 |
| ENSG00000075240 | *GRAMD4* | GRAM domain containing 4 | | 7 | 0 | 265 | 0 |
| ENSG00000183963 | *SMTN* | Smoothelin | | 24 | 0 | 125 | 0 |
| ENSG00000100234 | *TIMP3* | TIMP metallopeptidase inhibitor 3 | | 1 | 0 | 211 | 3 |
| ENSG00000196924 | *FLNA* | Filamin A | Chr X | 21 | 0 | 198 | 36 |
| ENSG00000126012 | *KDM5C* | Lysine demethylase 5C | | 15 | 0 | 171 | 11 |
| – | – | – | Chr Y | – | – | – | – |

| Chr | 1 | 2 | 3 | 4 | 5 | 6 | 7 | 8 | 9 | 10 | 11 | 12 | 13 | 14 | 15 | 16 | 17 | 18 | 19 | 20 | 21 | 22 | X | Y |
|---|---|---|---|---|---|---|---|---|---|---|---|---|---|---|---|---|---|---|---|---|---|---|---|---|
| ## | 6 | 2 | 3 | 1 | 3 | 2 | 1 | 1 | 1 | 2 | 3 | 2 | 1 | 1 | 2 | 2 | 1 | – | 2 | 1 | 1 | 4 | 2 | – |

[1] *Ortholog*: Gene that diverged upon evolutionary separation of species lineages.
*Paralog*: Gene derived from the same ancestor by duplication within the same species.
[2] Negative transcriptional regulator of basic helix-loop-helix (bHLH) transcription factors.

**Table S5.** Pharmaceuticals modifying the mRNA expression [1] of select regulome-assigned hepatic genes that display highly significant age-dependent differences in expression. Active pharmaceutical ingredients (APIs) that affect (A), decrease (↓) or increase (↑) the transcription of *PPP1R10*, *IGFALS* or *DUSP1* are listed in alphabetical order. The term, 'affects', indicates that the respective studies referred to in the Comparative Toxicogenomics Database generated ambiguous results. APIs interfering with the expression of ≥2 of the three regulome-assigned genes are highlighted by framing.

| **API** | **Regulome-assigned Genes** | | | | | | | | |
|---|---|---|---|---|---|---|---|---|---|
| | ***PPP1R10*** | | | ***IGFALS*** | | | ***DUSP1*** | | |
| | Out of 126 chemical interactions listed [2] | | | Out of 130 chemical interactions listed [3] | | | Out of 646 chemical interactions listed [4] | | |
| | **A** | ↓ | ↑ | **A** | ↓ | ↑ | **A** | ↓ | ↑ |
| Acetaminophen | + | + | – | + | + | – | + | + | – |
| Afimoxifene | – | – | – | – | – | – | – | – | + |
| Amphetamine | – | – | – | – | – | – | – | – | + |
| Ampicillin | – | – | – | – | – | – | – | – | + |
| Antirheumatic agents | – | – | – | – | – | – | – | + | – |
| Aspirin | – | – | – | – | – | – | + | – | – |
| Azathioprine | – | – | – | – | – | – | – | – | + |
| Cisplatin | – | + | – | – | + | – | – | + | – |
| Curcumin | – | – | – | – | – | – | – | – | + |
| Cyclosporine | – | + | – | – | – | + | – | – | + |
| Dexamethasone | – | – | – | – | – | – | – | – | + |
| Diclofenac | – | – | – | – | – | – | + | – | – |
| Doxorubicin | – | + | – | – | – | – | – | + | – |
| Fluorouracil | – | – | – | – | – | – | – | – | + |
| Folic acid | – | – | – | – | – | – | – | + | – |
| Gentamicins | – | – | + | – | – | – | – | – | – |
| Hydrocortisone | – | – | – | – | – | – | – | – | + |
| Indomethacin | – | – | – | – | – | – | – | – | + |
| Leflunomide | – | + | – | – | + | – | – | – | + |
| Lithium | – | – | – | – | – | – | – | – | + |
| Methimazole | – | – | – | – | – | – | – | – | + |
| Methotrexate | – | – | – | – | – | – | – | – | + |
| Methyltestosterone | – | – | – | – | – | – | – | + | – |
| Omeprazole | – | – | – | + | – | – | – | – | – |
| Penicillamine | – | – | – | – | – | – | – | + | – |
| Phenobarbital | + | + | – | – | – | – | – | – | – |
| Raloxifene hydrochloride | – | – | + | – | – | – | – | – | – |
| Tamoxifen | + | – | – | – | + | – | + | – | – |
| Testosterone | – | – | – | – | – | – | – | – | + |
| Thimerosal | – | – | – | – | – | – | – | – | + |

| | | | | | | | | | |
|---|---|---|---|---|---|---|---|---|---|
| Ursodeoxycholic acid | – | – | – | – | + | – | – | – | – |

[1] According to the Comparative Toxicogenomics Database, North Carolina State University, Raleigh, NC 27695-7617, USA. Source: http://ctdbase.org/ (accessed on 20 July 2021, which applies to all footnotes).
[2] Specifics for *PPP1R10*: http://ctdbase.org/detail.go?type=gene&acc=5514&view=ixn.
[3] Specifics for *IGFALS*: http://ctdbase.org/detail.go?type=gene&acc=3483&view=ixn.
[4] Specifics for *DUSP1*: http://ctdbase.org/detail.go?type=gene&acc=1843&view=ixn. .
